# The Therapeutic Outcome of Sialendoscopy in Patients with Sialoadenitis 

**DOI:** 10.22038/IJORL.2023.63433.3174

**Published:** 2023-03

**Authors:** Mohammad Farhadi, Saleh Mohebbi, Ahmad Daneshi, Mohammad Jafaripanah, Marjan Mirsalehi, Ali Omidvari

**Affiliations:** 1 *ENT And Head and Neck Research Center and Department, Hazrat Rasoul Hospital, The Five Senses Health Institute, School of Medicine, Iran University of Medical Sciences, Tehran, Iran.*; 2 *Skull Base Research Center, the Five Senses Institute, Iran University of Medical Sciences, Tehran, Iran.*

**Keywords:** Sialendoscopy, Sialadenitis, Surgery, Parotid gland, Submandibular gland

## Abstract

**Introduction:**

Recent advances have led to the development of sialendoscopy, an accurate, minimally invasive procedure with high diagnostic and therapeutic capabilities in treating sialolithiasis. This study aimed to evaluate the results and complications of sialendoscopy in patients suffering from sialoadenitis.

**Materials and Methods:**

This study was a prospective interventional case series study on patients with sialoadenitis due to sludge or stone formation preoperatively confirmed by sonography or computed tomography (CT) scanning. Diagnostic sialendoscopy was performed, and the presence of stenosis, sludge, or stones inside the gland or duct was examined, and surgery was done. During follow-up time (18.8 ± 7.4 months), recurrence of symptoms, the need for reoperation, and postoperative complications were also assessed.

**Results:**

The sialendoscopy was performed in 51 patients, including 55 glands. Forty-five Patients (88.2%) reported pain relief, and 46 patients (90.2%) reported that the treatment using sialendoscopy was better than conservative methods. The duct restenosis also occurred in one patient requiring open surgery. In assessing the main factors predicting the need for reoperation, the site of involvement (parotid versus submandibular glands) and the size of the stone were identified as the main determinants. The best cut-off value for stone size in predicting reoperation requirement was 7.0mm, with a sensitivity of 100% and a specificity of 85.7%.

**Conclusion:**

Intraoperative sialendoscopy is a successful diagnostic and therapeutic tool with minimal postoperative complications in salivary gland duct involvement patients.

## Introduction

Recent decades have been marked by clinical research in otolaryngology and head and neck surgery toward organ preservation and tissue function. Morbidity in head and neck diseases has been greatly reduced with the adoption of new methods, and open surgical procedures are largely replaced by endoscopic methods (1).

Although different suggested treatment protocols exist for salivary gland tumors, other pathologies, such as sialolithiasis and recurrent parotitis in adolescents, are treated as desired (2). Sialadenitis secondary to underlying obstructive pathology, including stenosis and duct polyps, is the most common disorder of the salivary glands (3). Patients suffering from sialadenitis are frequently scheduled for various therapeutic protocols, including medications (antibiotics, steroids, and anticholinergics) or surgical interventions such as intraoral incisions, sialolith removal, or even gland resection (4). Initial treatment of obstructive sialadenitis is usually conservative with fluid intake, salivation stimulation, and antibiotics used in suspected bacterial infections. In recurrent cases, surgical protocol (gland resection) is usually used (5,6). 

With the introduction of sialendoscopy, the management of salivary gland obstruction has undergone a dramatic transformation. Sialendoscopy was first used in 1991 to evaluate salivary gland ducts (7). 

Sialendoscopy is a minimally invasive procedure that involves a small-calibrated endoscope that facilitates direct examination of the salivary duct system. Sialendoscopy consists of rigid endoscopes designed to simplify diagnostic evaluation of the salivary ducts (8). By applying therapeutic sialendoscopy, the placement of wire baskets, micro forceps, lasers, and balloons can be facilitated and, in this regard, relieving duct obstruction such as in the background of sialolithiasis and duct stenosis can be successfully managed (9). In addition, rinsing the duct, injecting steroids, removing duct sludge, and relieving glandular inflammation can also be done (10).

However, there are still few reports on the usefulness of this method or its possible and potential side effects. This study aimed to evaluate our experience, therapeutic results, and complications of sialendoscopy in patients with inflammation and swelling of the parotid and submandibular salivary glands referred to our center. 

## Materials and Methods

This study was a prospective interventional case series study on patients with inflammation and swelling of the parotid and submandibular glands (sialadenitis) due to the formation of sludge in the salivary gland ducts because of stones, stenosis, or debris inside the salivary ducts. The diagnosis was preoperatively confirmed by sonography or computed tomography (CT) scanning. Patients with any tumor or pathological causes of salivary gland masses and any surgery on salivary glands were excluded from this study. The study was designed in a tertiary referral center, a university-affiliated hospital, and written consent from the patient was required to participate. 

After intubation and induction of anesthesia and within surgery, diagnostic sialendoscopy was performed, and the presence of stenosis, sludge, or stones inside the gland or duct was examined. In the cases of sialolithiasis, the stone was removed using wire baskets. If there was sludge in the examined duct, we removed the debris. In the cases of severe stenosis, dilation and stent insertion were done for the patients. For all patients, irrigation with normal saline was done. The patients were monitored in the hospital for 24 hours after surgery. They were then discharged with antibiotics for up to a week. Patients were finally evaluated the day after surgery and one month and three months after surgery with a clinical examination of the surgical site regarding erythema, edema, pain and improvement of swelling. The recurrence of symptoms and infection, the need for reoperation, and postoperative complications were also assessed during the follow-up time. 

For statistical analysis, results were presented as mean ± standard deviation (SD) for quantitative variables and were summarized by frequency (percentage) for categorical variables. Continuous variables were compared using the t-test or Mann-Whitney U test whenever the data did not appear to have normal distribution or when the assumption of equal variances was violated across the study groups. Categorical variables were, on the other hand, compared using the chi-square test. The receiver operating characteristic (ROC) curve analysis was used to determine the cutoff value of stone size in predicting the requirement of reoperation. The statistical software SPSS version 19 for windows (IBM, Armonk, New York) was used for the statistical analysis. 

## Results

*General information*

A total of 51 patients underwent sialendoscopy during the study. Of these 51 patients, 28 (54.9%) were male, and 23 (45.1%) were female. The mean age of the patients at the time of surgery was 39.3 ± 16.8 years (ranged 3 to 75 years). The mean duration of the disease symptoms was 14.7 ± 13.6 months (ranged 1 to 60 months). Multiple glandular involvements were revealed in 4 patients (Bilateral parotids involvement in three patients and bilateral submandibular glands in another one). Diagnostic endoscopy was successful in all cases. Table 1 summarizes clinical manifestations and physical examination details.

**Table 1:** clinical manifestations and physical examination at presentation

**clinical manifestations**		**physical examination**	
swelling of the gland	50 (98%)	swelling of the gland	50 (98%)
pain	50 (98%)	tenderness	12 (23.5%)
eating-induced pain	27 (54.0%)	stone in two-handed palpitation	9 (18.0%)
history of fever	4 (7.8%)	erythema	1 (2%)
intraoral secretions of pus	3 (5.9 %)		

*Preoperative evaluations*

Therapeutically, 80.0% of patients underwent conservative therapies, including antibiotics, analgesics, sialogogues, and warm compression, leading to temporary improvement of the symptoms in all patients, but recurred after a few days or weeks. All cases were assessed by ultrasonography, which led to the diagnosis of sialolithiasis, and 18 patients (43.9%) were also evaluated by CT scan. The mean size of calculi was 5.2 ± 2.2 mm (ranged 3 to 10 mm). Ductal dilatation was also observed in 35 patients (85.3%). In thirteen cases, gland removal was planned before referring to our department.

*Sialendoscopy findings:*

Sialendoscopy was performed on 51 patients, including 55 glands. In this regard, No intraoperative complication was noted. Table 2 summarizes the pathological findings according to underlying disease conditions in patients undergoing sialendoscopy.

**Table 2:** The final diagnosis of the patients who underwent sialendoscopy

	**Chronic recurrent sialadenitis**	**Sialolithiasis**	**Sjogren’s syndrome**	**Juvenile recurrent parotiditis**	**Ductal cyst**	**Total**
Submandibular gland	13	8	2	0	0	23
Parotid gland	16	13	0	2	1	32
Total	29	21	2	2	1	55

*Sialolithiasis*

**Fig 1:**
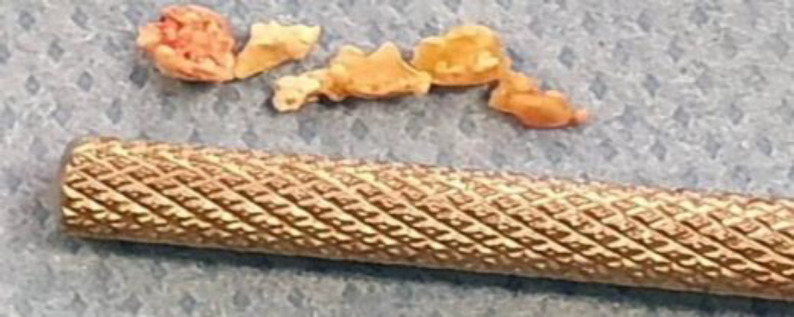
Removed stones in a patient with multiple stones in Wharton duct

**Fig 2:**
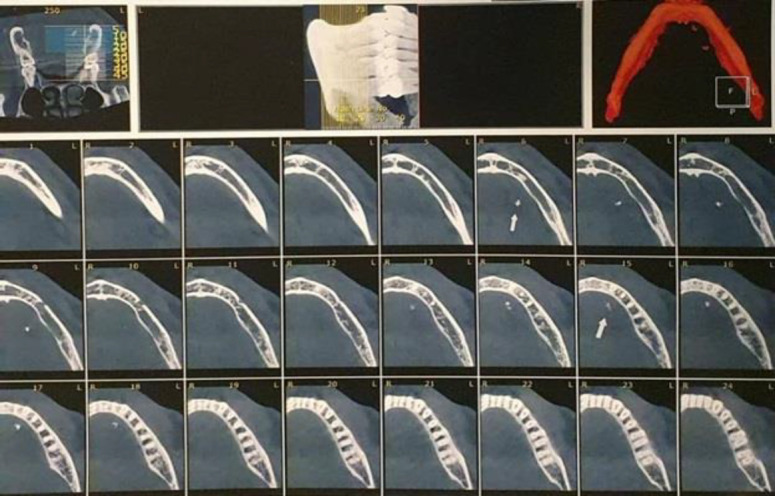
Computed tomography showing Wharton duct multiple stones

A single stone was revealed in 21 glands (38.2%). Multiple stones (ranged 2 to 6 stones) were reported in 5 patients (Figures 1 and 2). 

In two cases, a small stone came out just with repeated irrigation. Two cases needed an open approach because of the size and proximal position of the calculi. 

In two cases, a small stone came out just with repeated irrigation. Two cases needed an open approach because of the size and proximal position of the calculi. 

*Ductal stenosis*

Ductal stenosis was found in 32 patients (62.7%), which needed dilatation in 3 patients (5.9%). In seven cases of single distal stenosis, a costume-made stent was used. The stents stayed in place for one month. 

*Postoperative follow-up*

Concerning postoperative consequences, the mean follow-up time was 18.8 ± 7.4 months (11 -43 months). The first follow-up visit was one week after surgery and every three months for a year. Forty-five Patients (88.2%) reported pain relief, and 46 patients (90.2%) were satisfied with endoscopic treatment versus conservative methods. Nineteen patients experienced a mild increase in postoperative swelling which resolved within 3.5 ± 1.4 days (ranged 2 to 7 days). Postoperative bleeding at the site of surgery, tongue numbness, and facial paralysis did not occur postoperatively. The swelling of the glands was resolved a few months after surgery in 50 of 55 glands (90.9%). The postoperative complications, including sialocele and fistula, occurred in none of the patients, but infection and intraoral pus drainage also occurred in 3 patients (5.4%); 2 resolved with antibiotic, and the next one underwent superficial parotidectomy.

In total, three cases needed open surgery, which was parotidectomy. The first one was large (8 mm) and stuck in the proximal part of the parotid gland. The second one required open surgery due to ductal restenosis because of multiple stenoses, the fibrosis of the ducts, and the presence of a ductal cyst. In two of these cases was advised to remove the gland before sialendoscopy, and we performed sialendoscopy as an initial procedure.

*Factors predicting the need for reoperation*

In assessing the main factors predicting the need for reoperation, the site of involvement (parotid versus submandibular glands) and the size of the stone were identified. In this regard, the need for reoperation was found in 20.0% of parotid glands and none of the submandibular glands (p = 0.026). Also, the mean size for stones with and without requiring reoperation was 9.00±1.14mm and 4.61±1.73mm, respectively (p = 0.003). Finally, ROC curve analysis was performed to predict the need for reoperation regarding the stone size. The best cut-off point for stone size to predict needing reoperation was 7.0 mm, with a sensitivity of 100% and a specificity of 85.7%. (Area under the curve: 0.955).

## Discussion

Sialolithiasis is a common major salivary gland disorder frequently affecting the submandibular gland and, less commonly, the parotid or sublingual glands. Although ultrasonography has been accepted as a gold standard diagnostic tool to detect large salivary calculi (higher than 1.5cm in diameter), smaller stones may face many false negatives (3). Other imaging tools that can help to reveal salivary calculi include plain sialography, computed tomography (CT), scintigraphy, or even magnetic resonance sialography (4). We evaluated all cases with ultrasound, and 44 percent with CT-scan for better assessment. CT-scan could be helpful for better surgical planning and preparation for a different or difficult situation.

However, recent advances have led to the development of a new technique, sialendoscopy, as an accurate, minimally invasive procedure with proper diagnostic and therapeutic capabilities. Some authors have claimed the accuracy of 100% for this procedure for detecting salivary calculi with different sizes and opacity (10). In the cases without proper response to conservative management, the use of sialendoscopy as a treatment approach has been accompanied by good treatment outcomes with minimal postoperative complications. This tool is now accepted as a good alternative for surgical interventions, especially in cases with small stones (<5mm). In larger calculi, applying sialolithotripsy followed by sialendoscopy has been introduced as a choice (11,12). According to various reports, the overall success rate of sialendoscopy in treating sialolithiasis is high, in the range of 85 to 90% (13,14). As shown in the present study and consistent with the previous observations, in a long-term following-up (about 18.8 months), the success rate was found to be 88.2 % with the approach to relieving pain and 90.2% to relieve calculi. Additionally, postoperative complications following this procedure were low and mostly reversible and tolerable. We also noted no major complications. Moreover, requiring reoperation due to post-procedural sequels remained rare. Thus, based on our findings, diagnostic followed by therapeutic sialendoscopy can be accepted as the first choice for treating sialolithiasis and most salivary glandular duct pathology. 

In a systematic review of 13 trials by Galdermans et al. in 2020 (15), the success rate of sialendoscopy in cases suffering parotid sialolithiasis was 71.4% to 100% with no major postoperative complications, which is consistent with the results of our study. As Matsunobu et al. in 2014 (16) indicated, using sialendoscopy led to treating sialolithiasis in 57.5% with the ultimate need to remove the submandibular gland removal in 13.7%. 

The only factors responsible for their partially lower success rate included considering large stones (up to 20mm). In another study by Cox et al. in 2018 (17), sialendoscopy successfully found all sialolithiases. 

In their study, a stone size of less than 5 mm and a distance from the papilla of less than 3 cm were the main determinants for procedure success. Also, Zenk et al. in 2012 showed that the diagnostic accuracy of sialendoscopy was about 90% (18), with a required submandibular and parotid glands removal rate of 4% and 4%, respectively. Similarly, our procedure was successful in 100 percent of diagnoses and 94 percent in endoscopic treatment. Saving 11 glands out of 13 cases that advised removing the gland was a great success (84%). 

Therefore, by ruling out the candidate for surgical approaches (due to large stone sizes), sialendoscopy can be considered a choice for treating sialolithiasis. 

## Conclusion

As a noninvasive method, it can be concluded that sialendoscopy successfully treats sialolithiasis with an overall success rate of 90.2%, leading to minor and reversible postoperative complications. 

In other words, such a procedure can be a suitable therapeutic option for treating small salivary gland calculi (smaller than 7mm); however, further studies should be performed to determine the cut-off value of stone size for selecting sialendoscopy as the therapeutic procedure instead of surgical interventions as well as the main factors predicting successfulness of such procedure. 
